# Small intestinal bacterial overgrowth in systemic sclerosis: a review of the literature

**DOI:** 10.1007/s00403-018-1874-0

**Published:** 2018-10-31

**Authors:** Beata Polkowska-Pruszyńska, Agnieszka Gerkowicz, Paulina Szczepanik-Kułak, Dorota Krasowska

**Affiliations:** Chair and Department of Dermatology, Venerology and Paediatric Dermatology, Medical University of Lublin, Lublin, Poland

**Keywords:** Systemic sclerosis, Small intestinal bacterial overgrowth, SIBO, Diagnosis, Therapy

## Abstract

Systemic sclerosis (SSc) is a chronic, connective tissue disease with an autoimmune pattern characterized by inflammation, fibrosis and microcirculation changes leading to internal organs malfunctions. Recently, the presence of uncharacteristic gastrointestinal symptoms in the course of SSc has been underlined. The possible cause of such clinical presentation is the small intestinal bacterial overgrowth (SIBO). Nevertheless, these manifestations resulting from gastrointestinal tract hypomotility may occur in numerous disease entities. The systematic review of the literature was performed on MEDLINE database using the relevant MeSH terms including all sub-headings. After further investigation, the initial number of 56 records was limited to 7 results. The study analysis showed an increased presence of SIBO in 39% of patients suffering from SSc. The average SSc duration was longer in SSc patients with coexisting SIBO. SIBO remains a diagnostic and therapeutic challenge and therefore is a significant clinical problem among patients suffering from SSc.

## Introduction

Systemic sclerosis (SSc) is a chronic, multisystem connective tissue disease of the autoimmune etiology characterized by the microcirculation changes, skin and internal organs fibrosis and the presence of autoantibodies. These particular pathogenetic processes lead to cutaneous lesions as well as systemic complications [1, 3, 6, 8]. In genetically predisposed individuals certain factors may cause endothelial cell injury triggering the production of cytokines activating T- and B-lymphocytes. B-lymphocytes produce autoantibodies against gastrointestinal smooth muscles muscarinic receptors altering neuro-muscular junction function. Along with the fibrosis of the gastrointestinal tract provoked by interleukins 4, 6, and 13 produced by Th2 lymphocytes as well as tumor growth factor β produced by macrophages these lead to gastrointestinal hypomotility [17]. Approximately 90% of SSc patients present variously intensified fibrosis in the gastrointestinal tract [3]. Autopsy studies showed muscle atrophy and/or fibrosis in the esophagus, small intestine and large intestine in 74, 48, and 39%, cases, respectively [8]. It is estimated that gastrointestinal involvement is responsible for around 11% of deaths within SSc patients.

Apart from fibrosis, an important clinical problem is the malfunction of the gastrointestinal tract, including particularly small intestine impairment and affecting around 8–50% of patients [7, 8, 24]. Usually, patients suffer from bloating, early satiety, periodic diarrhea or food intolerance, most commonly lactose intolerance. Intestinal complications may lead to severe absorption disorders and malnutrition, which significantly aggravate the prognosis of patients [7, 8, 24]. The bowel disorders can be possibly caused by the small intestinal bacterial overgrowth (SIBO). Although the gastrointestinal involvement in systemic sclerosis has been well established, there are few literature data on clinical characteristics of SIBO and its prevalence in SSc patients. The aim of this study was a systematic review of the literature data regarding SIBO presence in patients with systemic sclerosis.

## SIBO—definition and clinical symptoms

The human gastrointestinal tract is colonized by numerous microorganisms, which, depending on the localization, vary both quantitatively and qualitatively. The proximal small intestine consists a small number of bacteria, estimated at the maximum of 10^3^ CFU/ml (colony forming units), whereas in the large intestine the number of bacteria reaches 10^4^ CFU/ml [5, 11, 13, 20]. The maintenance of the normal microbiota environment is very important and every abnormality in this area may lead to serious disorders. One of the possible complications can be the small intestinal bacterial overgrowth, defined as the increase in the number of bacteria to over 10^5^ CFU/ml or as the presence of atypical flora. Multiple mechanisms are preventing abnormal and extensive bacterial growth. They include gastric acid secretion, bacteriostatic properties of pancreatic juice and bile, the presence of secretive immunoglobulins (SIgA) on the mucous surfaces, vivid intestinal peristalsis and adequately functioning ileocecal valve (Bauchin’s valve) [13]. The imbalance of these defence mechanisms, anatomical abnormalities or intestinal motility disorders may lead to SIBO development.

The possible connection between SIBO and SSc seems very interesting. However, the literature data regarding the presence of SIBO in SSc patients have not been widely studied yet and needs further investigation [7]. The impaired gut motility is considered to be one of the leading causes of SIBO in the course of SSc. It may also lead to another SIBO risk factor—the chronic pseudo-obstruction. Moreover, the SSc patients receiving proton pump inhibitors in the therapy of increased gastric acid secretion may also suffer from the disruption of the defence mechanisms preventing the excessive bacterial colonization of small bowel [5, 7, 20]. The symptoms of SIBO including their pathogenesis are depicted in Table 1 [5, 11, 13, 20].

**Table 1:** The causes of small intestinal bacterial overgrowth symptoms [5, 11, 13, 20]

Symptoms	Pathogenesis
Flatulence	Bacterial fermentation of carbohydrates with water, short-chain fatty acids and gases overproduction in intestinal lumen
Diarrhea	
Steatorrhoea	Bacterial deconjugation of bile acids leading to insufficient absorption of fats and fat-soluble vitamins
Fat-soluble vitamins deficiency symptoms (A, D, E)	
Neurological and psychiatric symptoms of malignant anaemia	Bacterial vitamin B12 consumption
Malabsorption syndrome symptoms (weight loss, no weight gain, malnutrition)	Reduced availability of nutrients (proteins, sugars) used by bacteria
Hypoproteinemia symptoms (symmetrical, pitting oedema)	Impaired function of the intestinal barrier causing increased protein permeability and protein loss
Systemic disorders (glomerulonephritis, hepatitis, fatty liver disease, arthritis, tendonitis) and cutaneous lesions	Increased bacterial counts and intestinal barrier destruction lead to the development of antigenemia and consequently to the production of antibodies and the development of type III hypersensitivity reactions

## The prevalence of SIBO in SSc—the systematic review of the literature

### Materials and methods

**Fig. 1:**
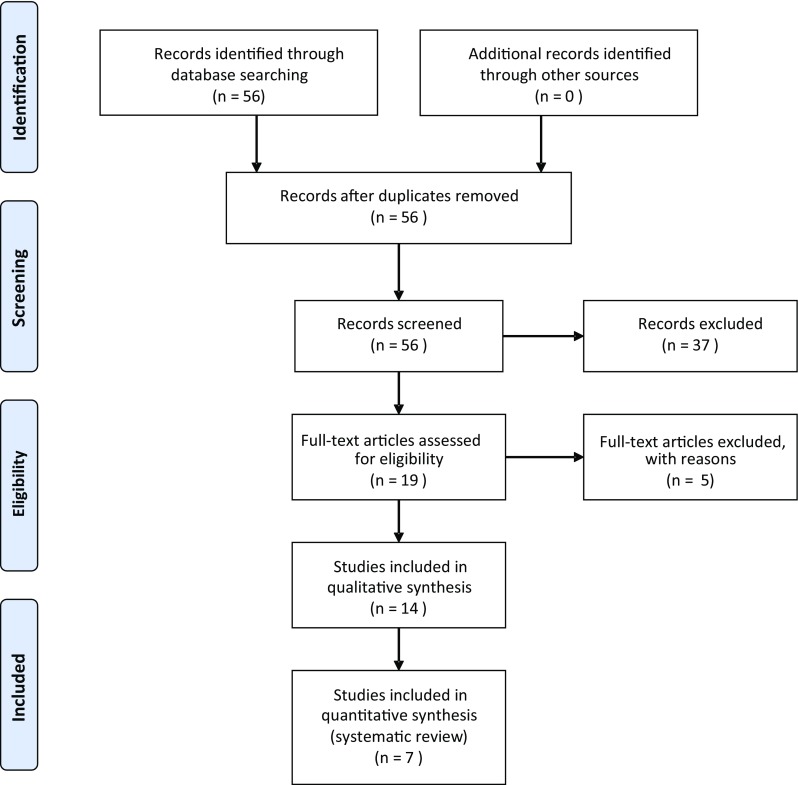
The systematic review was prepared according to PRISMA statement

The systematic review of the literature was performed on MEDLINE database from 2007 to 2017 complementary to PRISMA protocol (Fig. 1). The inclusion criteria for considering studies for the review based on PICOS structure comprised the population of adult patients diagnosed with systemic sclerosis, performing the hydrogen breathing test as the intervention, preferably the presence of healthy group control, outcomes measured in parts per million in hydrogen breathing test and the included type of studies were retrospective and prospective clinical studies, cohort studies as well as one-case series.

The date of the last search was April 2017. The database was searched using the relevant MeSH terms including all sub-headings. The studies reporting the prevalence of SIBO in SSc patients were identified from the database by utilizing the search terms scleroderma OR systemic sclerosis AND small intestinal bacterial overgrowth OR small intestine bacterial overgrowth OR SIBO OR small bowel bacterial overgrowth OR small intestine overgrowth OR small intestinal overgrowth. The initial search revealed 56 results. After applying additional criteria (English language publications, human studies), the database search revealed 19 records. Five publications describing other than gastrointestinal involvement were excluded. After further investigation, we selected retrospective and prospective clinical studies, cohort studies as well as one-case series study receiving seven results. The studies included in the review are presented in Table 2 [8, 10, 12, 19, 21, 23, 24].

**Table 2:** The prevalence of small intestinal bacterial overgrowth in patients with systemic sclerosis [10, 12, 18, 19, 21, 23, 24]

Study	Marie et al. [19]	Savarino et al. [23]	Fynne et al. [10]	Gemignani et al. [12]	Tauber et al. [24]	Parodi et al. [21]	Marie et al. [18]
Study duration	01.2011–06.2014	no data	no data	06.2009–03.2011	01.2011–12.2012	No data	01.2006–06.2007
Study group (SSc^a^ patients)	*n* = 125	*n* = 99	*n* = 15	*n* = 50	*n* = 37	*n* = 55	*n* = 51
Women/men	99/26	80/10	13/12	43/7	29/8	50/5	41/10
Average age (years)	55	59	58	54	60	59	54
Number of patients with dcSSc^b^	43 (34.4%)	31 (31%)	15 (100%)	18 (36%)	14 (38%)	18	25 (49%)
Number of patients with lcSSc^c^	82 (65.6%)	68 (68%)	0	32 (64%)	23 (62%)	37	26 (51%)
Average duration of the disease (years)	4	87% > 5 12% < 5	No data	5	9	No data	4
Control group of healthy people	No control group	*n* = 60	*n* = 17	*n* = 60	No control group	*n* = 60	No control group
Women/men	–	50/10	12/5	48/12	–	48/12	–
Average age (years)	–	57	52	48	–	51	–
Diagnostic methods for SIBO^f^	GHBT^d^	LHBT^e^	GHBT	GHBT	GHBT	LHBT	GHBT
Criteria for positive diagnostic test result	(1) H_2_^g^ and/or CH_4_^h^ increase > 20 ppm^i^ above basal value; (2) H_2_ and/or CH_4_ increase > 10 ppm on 2 consecutive measurements within the 2 first hours;( 3) H_2_ and/or CH_4_ increase > 10 ppm between minimal and maximal values	H_2_ and/or CH_4_ excretion increase > 10 ppm compared with baseline in three consecutive air samples	H_2_ and/or CH_4_ increase > 10 ppm above basal value	H_2_ and/or CH_4_ increase > 12 ppm above basal value	H_2_ and/or CH_4_ increase > 20 ppm in 2 consecutive measurements H_2_ and/or CH_4_ increase > 12 ppm in 3 consecutive measurements	H2/CH4 excretion increase > 10 ppm compared to the basal value in 2 consecutive measurements	(1) H_2_ and/or CH_4_ increase > 20 ppm above basal value; (2) H_2_ and/or CH_4_ increase > 10 ppm on 2 consecutive measurements within the 2 first hours; (3) H_2_ and/or CH_4_ increase > 10 ppm between minimal and maximal values
Positive results for SIBO, *n*							
In the study group	44 (46,2%)	47 (46%)	3 (21%)	9 (18%)	14 (38%)	30 (55%)	22 (43%)
In the control group		3 (5%)	No data	3 (5%)		4 (7%)	
Type of study	Prospective	Retrospective cohort	Prospective cohort	Prospective cohort	Prospective	Prospective cohort	Case series

^a^Systemic sclerosis

^b^Diffuse systemic sclerosis

^c^Limited systemic sclerosis

^d^Glucose hydrogen breath testing

^e^Lactulose hydrogen breath testing

^f^Small intestinal bacterial overgrowth

^g^Hydrogen

^h^Methane

^i^Parts per million

## Results

The study analysis showed the presence of SIBO in around 39% (18–55%) patients suffering from SSc [10, 12, 18, 19, 21, 23, 24].

The average SSc duration was longer in patients with SIBO diagnosis, on average by 3.7 years longer, while the data on the existence of SIBO depending on the age of the patients remain uncertain. No connection was found between the SIBO occurrence and the subsets of systemic sclerosis (diffuse SSc or limited SSc). The antibodies against topoisomerase I (Scl-70) were less frequent in SIBO patients [24]. Whereas anti-centromere antibodies were present with similar frequency both in the group of patients with SIBO and without SIBO (Table 3) [18, 24].

**Table 3:** The laboratory finding in patients with systemic sclerosis, depending on the occurrence of SIBO syndrome [18, 24]

	Marie et al. [18]			Tauber et al. [24]		
	Patients with SIBO^a^ (*n* = 22)	Patients without SIBO (*n* = 29)	*p* value	Patients with SIBO (*n* = 14)	Patients without SIBO (n = 23)	*p* value
Clinical characteristic						
SSc^b^ duration (years)	8.3 (1–37)	4.9 (1–20)	0.0067	11 (1–29)	7 (3–35)	0.02
Age (years)	59.5 (23–82)	50 (34–73)	0.0292	61.5 (42–80)	59 (35–79)	0.5
SSc subset, *n* (%)						
dcSSc^c^ lcSSc^d^	8, (36.4) 14, (63.6)	17 (58.6) 12 (41.4)	0.159	5 (36) 9 (64)	9 (39) 14 (61)	0.9
Laboratory findings						
Anti-Scl70 Ab^e^	22.7%	27.6%	0.755	7% (1)	39% (9)	0.04
ACA Ab	40.9%	24.9%	0.235	57% (8)	33% (7)	0.3
Hemoglobin (g/dl)	12.2 (8.9–14.5)	13.9 (10.3–15.5)	0.002	No data		
Ferritin (µg/l)	44.5 (5-307)	60 (2-730)	0.361	51.9 (10–147)	63.6 (10–170)	0.07
Vitamin B12 (pmol/l)	225 (30–748)	288 (131–587)	0.133	322 (166–697)	373 (232–488)	0.1
Total serum protein (g/l)	65.5 (51–77)	69 (55–76)	0.66	No data		
Serum albumin (g/l)	39 (32–49)	42 (30–50)	0.024	39.2 (35–44)	40 (33–45)	0.2
Phosphor (mmol/l)	No data			1.05 (0.83–1.35)	1.21 (0.94–3.32)	0.03
Calcium (mmol/l)	No data			2.27 (2.14–2.41)	2.33 (2.22–2.47)	0.03
Triglycerides (mmol/l)	No data			0.96 (0.66–1.24)	1.51 (0.64–3.32)	0.04
ESR^f^ (mm/h)	24 (4–70)	8 (2–78)	0.003	No data		

^a^Small intestinal bacterial overgrowth

^b^Systemic sclerosis

^c^Diffuse systemic sclerosis

^d^Limited systemic sclerosis

^e^Antibodies

^f^Erythrocyte sedimentation rate

The laboratory findings in patients with SIBO showed lower median levels of hemoglobin, ferritin, total serum protein, phosphor, calcium, and triglycerides and more elevated erythrocyte sedimentation rate in comparison with the group of patients without SIBO. The observations on serum albumin levels are unclear (Table 3) [22, 23].

Among the SSc patients the most characteristic clinical pattern included symptoms such as diarrhea, constipation, flatulence, abdominal pain, abdominal tenderness, nausea, vomiting, dysuria, tenesmus, dysphagia, reflux, weight loss and early satiety (Table 4) [10, 12, 18, 21, 24].

**Table 4:** Percent of patients with systemic sclerosis presenting selected gastrointestinal symptoms [10, 12, 18, 21]

	Marie et al. [18]		Parodi et al. [21]		Fynne et al. [10]	Gemigani et al. [12]
	Patients with sibo^a^ (n = 22)	Patients without SIBO (n = 29)	Patients with SIBO (n = 30)	Patients without SIBO (n = 25)	No group division	No group division
Diarrhea	50%	10.3%	~ 27%	~ 9%	50%	22%
Abdominal pain Upper Lower	86.4%	31%	− 30% − 34%	− 34% − 29%	50%	− 58% − 70%
Bloating	77.3%	44.8%	~ 57%	~ 50%	60%	62%
Constipation	59.1%	3.4%	No data		33%	46%
Nausea	54.5%	37.9%	~ 27%	~ 38%		52%
Vomiting	18.2%	3.4%	~ 4.5%	~ 3%	No data	20%
Abdominal tenderness	54.5%	6.9%	~ 54.5%	~ 46%	No data	40%
Fever	18.2%	0	0	0	No data	10%
Tenesmus	13.6%	0	~ 50%	~ 46%	40%	4%
Reflux	No data		No data		93%	No data
Dysphagia	No data		No data		33%	44%
Early satiety	No data		No data		25%	No data

^a^Small intestinal bacterial overgrowth

## Diagnostics

Despite numerous research, SIBO remains a clinically significant problem. Frequently patients with disorders falling within the spectrum of SIBO symptoms are unsuccessfully diagnosed. The causative factors are the lack of ideal diagnostic test and the insufficient standardization of the available diagnostic procedures [4, 14, 19, 22].

Screening the patients for SIBO should always be considered within patients with non-specific dyspeptic symptoms, motility disorders, gastrointestinal anatomical abnormalities, malnutrition or malabsorption [2, 5, 22]. The clinical manifestations may be a valuable hint; however, because of their low specificity and sensitivity they should not be taken into consideration as a sufficient diagnostic tool. It has been shown that the incidence of dyspeptic symptoms was similar in patients both with positive as well as negative hydrogen breathing test [16, 22].

### Small intestinal aspiration and culture

Despite high sensitivity, the culture of aspirated jejunum fluid is only a partially validated diagnostic method [15]. There is no full agreement on the number of bacteria in the small intestine that would define SIBO. However, it is assumed that bacterial count ≥ 10^3^ (CFU)/ml (colony forming units) is a significant value, and bacterial count ≥ 10^5^ CFU is an equivalent of SIBO diagnosis [22]. Unfortunately, there are some limitations to this method including invasiveness, time-consumption, high technical requirements, the lack of standardization of transport and culture methods [13] as well as the possibility of false-negative results in case of the endoscopic aspiration of the material only from the proximal part of small bowel [5, 13]. Kaye et al. used this method in SIBO diagnosis in SSc patients with 30% positive results [9]. At present, in SIBO diagnostics in SSc patients, it is highly recommended to perform additional tests.

### Glucose hydrogen breathing test (GHBT) and lactulose hydrogen breathing test (LHBT)

Significant progress in SIBO diagnostics was Erdogan et al. study, which compared the duodenal aspirate culture and glucose hydrogen breathing test in the group of 139 patients. The microbiological examination revealed SIBO presence in 45% of patients, whereas the breathing test was positive in 27% of patients. GHBT had lower sensitivity then duodenal aspiration and culture, whereas its specificity proved to be good. Considering the simplicity, low costs, easy access and non-invasiveness of this method, hydrogen breathing test should be used as a first line diagnostic test [9].

Most commonly used substrates in breathing tests are glucose and lactulose. The breathing tests use the particular ability of bacteria located in the small intestine to metabolize the carbohydrates to hydrogen and methane. These gases are absorbed in the intestinal wall and transported to the lungs where they are eventually excreted by the patient, which allows their detection in exhaled air. Characteristic changes of hydrogen or methane concentration in subsequent breath samples indicate the presence of SIBO. Before the breathing test, the patients should not consume any food for 12 h, for 48 h maintain low-fiber diet and avoid laxatives and antibiotics. At least for 3 h after oral glucose or lactulose administration, the measurements of hydrogen and methane concentrations in exhaled air samples are made every 15 min. The results processed by gas chromatography are expressed in ppm (parts per million) [14, 22, 24]. The definite result criteria for both GHBT and LHBT have not been adequately validated yet [10, 12, 18, 19, 21, 23, 24]. Hydrogen breathing tests were the most commonly used diagnostic tools in SSc patients in the performed systematic review (Table 2). However, they are difficult to compare with each other as different criteria were used to evaluate positive results.

### Evaluation of treatment efficiency

The clinical symptoms relapse is the measure of effectiveness and clinical response to empirical SIBO treatment. The lack of standardization regarding the type, dose, and duration of antibiotic therapy used puts, however, some serious limitations to this method. Furthermore, the potential adverse effects of empirical antibiotic treatment, the promotion of antibiotic-resistant bacterial strains and the development of pseudomembranous colitis have to be taken into account [10, 12, 19].

### Other diagnostic methods

Marie et al. evaluated the correlations between SIBO and abnormal calprotectin values in feces in SSc patients. The association of elevated fecal calprotectin levels has been reported in SSc patients with SIBO diagnosed with GBHT. Therefore, fecal calprotectin level can be useful in SIBO assessment in patients suffering from SSc [19]. Other methods include the evaluation of short-chain fatty acids in the small intestine aspiration, unconjugated bile acids in serum, urinary excretion of *p*-aminobenzoic acid or indican, and a 72-h test for fecal fat. Nevertheless, none of these methods are routinely used in clinical practice, and their usefulness has not been clearly defined [5, 14].

## SIBO treatment

SIBO therapy must be comprehensive and targeted to all causes, symptoms and complications [5]. The treatment options include metronidazole, amoxicillin with clavulanic acid, cotrimoxazole, ciprofloxacin, norfloxacin and rifaximin [20]. Therapeutic strategy according to EULAR recommendations involves the oral administration of amoxicillin during the first month (500 mg 3×/24 h), ciprofloxacin during the second month (500 mg 2×/24 h) and metronidazole during the third month (500 mg 3×/24 h). 43% of SSc patients diagnosed with SIBO showed beneficial effects of such therapy [24]. Currently, high expectations are pinned on rifaximin, nonabsorbable in the digestive tract and presenting bactericidal activity. The effectiveness of rifaximin among SSc patients was estimated at the level of 73.3%. Particularly noteworthy is the complete cessation of diarrhea, the facilitation of other abdominal symptoms and the normalization of lactulose hydrogen breathing tests in all patients after rifaximin administration [21]. Due to the presence of the intestinal motility disorders, it is recommended to avoid the peristalsis reducing drugs such as anticholinergics or opioids in patients with SSc and SIBO [18].

Patients allergic to antibiotics or not responding to optimal doses may use an elemental diet as an alternative. It consists of substances easily digested and absorbed in the proximal small intestine, what prevents the delivery of nutrients to bacteria residing in the distal bowel. The beneficial effects of herbs, prebiotics and probiotics are also suggested [20, 22]. Nevertheless, there is no data on the use of such therapy in SSc.

## Conclusions

Small intestinal bacterial overgrowth remains a significant clinical problem among patients suffering from systemic sclerosis. The presence of symptoms such as dyspepsia, flatulence, diarrhea, absorption disorders and malnutrition indicates the necessity of differential diagnosis towards small intestinal bacterial overgrowth.

The SIBO therapy should comprise the treatment of the symptoms and complications, a sufficient and adequate diet and cyclic antibiotic therapy. It is essential to eliminate the risk factors of SIBO, to treat the primary disease, and to neutralize the gastrointestinal motility disorders. Regrettably, hypomotility and fibrosis in the course of SSc are the final consequences of the pathophysiological processes involving the gastrointestinal tract and in most patients are irreversible. At present, there are no efficient therapies that could reverse the fibrosis of the gastrointestinal tract. The lack of an ideal diagnostic tool underlines the need for the search of new tests and biomarkers that will enable to establish a confident diagnosis of SIBO.
